# Transgenerational transfer of genocidal trauma: a systematic review and meta-analysis

**DOI:** 10.3389/fpsyt.2025.1699835

**Published:** 2026-01-30

**Authors:** Larysa Zasiekina, Iryna Hlova, Oleg Kokun, Illia Kuznietsov, Tetiana Pastryk, Olena Solonenko, Serhii Zasiekin

**Affiliations:** 1 University of Exeter, Exeter, United Kingdom; 2 Lesya Ukrainka Volyn National University, Lutsk, Ukraine; 3 G. S. Kostiuk Institute of Psychology, Kyiv, Ukraine; 4 Purdue University, West Lafayette, IN, United States; 5 Volyn Medical Institute, Lutsk, Ukraine; 6 University College London, London, United Kingdom

**Keywords:** genocide survivor, genocidal trauma, transgenerational transfer of trauma, posttraumatic stress disorder, trauma-related mental health disorders

## Abstract

This meta-analysis aims to estimate the prevalence of posttraumatic stress disorder (PTSD) among the offspring of genocide survivors and examine study-level moderators influencing PTSD rates across cultural settings. Following PRISMA guidelines, seven peer-reviewed studies were identified through systematic searches of major databases, covering genocides in Rwanda, Cambodia, Armenia and Israel (Holocaust). A total of 1,569 participants were included. Data were extracted on sample size, mean age, gender composition, generation, diagnostic assessment tools, and World Bank country classification. A random-effects model was used to estimate pooled prevalence, and meta-regressions assessed the moderating effects of gender composition and mean age. Heterogeneity was evaluated using I² and Q statistics. The pooled probable PTSD prevalence across the seven studies was 17.9% (95% CI: 9.9%-25.8%), which is significantly higher than global population norms during peacetime and not substantially lower than prevalence estimates reported in civilian populations living in war zones. Heterogeneity was substantial (I² = 95.03%, p <.001). Meta-regressions found no significant moderating effect of gender composition or age, possibly due to the limited number of studies with complete data and variations in study methodology. Overall, these findings underscore the persistent impact of genocidal trauma, highlighting how its effects extend beyond directly exposed individuals to shape the mental health of subsequent generations.

**Systematic review registration:**

https://www.crd.york.ac.uk/PROSPERO/view/CRD420251046525.

## Introduction

### Background and context

Over the past decades, the global landscape has witnessed an alarming increase in violent conflicts, encompassing wars, armed struggles, and acts of mass violence, including genocide (1, 2). Genocide, defined as systematic and deliberate attempts to annihilate specific ethnic, racial, religious, or national groups, constitutes one of the most severe forms of collective trauma due to its scale, intentionality, and lasting repercussions (3).

Historical and contemporary examples – such as the Holocaust, the Rwandan genocide, the Armenian genocide, the Bosnian genocide (e.g., Srebrenica), and the Cambodian genocide under the Khmer Rouge – highlight genocide’s devastating impacts on both individuals and communities (4–6). Some of these atrocities have been formally recognised, with perpetrators held accountable and survivors receiving public acknowledgements, reparations, or forms of justice and repentance (e.g., the Holocaust and the Rwandan genocide). However, even when genocide is officially recognised, its psychological consequences remain profound and enduring. Research consistently demonstrates elevated rates of PTSD and depression among Holocaust survivors, persisting for decades after the original trauma exposure (7, 8).

In contrast, other genocides remained hidden, denied, or politically silenced for decades. A striking example is the Holodomor – the man-made famine in Ukraine in 1932-1933, which is increasingly recognised as a genocide orchestrated by the Soviet government to break the will of the Ukrainian nation (9, 10). The long suppression of the truth surrounding such atrocities hinders collective healing and the justice aftermath of genocide (11, 12).

These catastrophic events not only cause immediate trauma for those directly affected but also leave deep psychological scars that extend beyond the initial victims, contributing to additional layers of intergenerational trauma (3, 6, 13). Survivors of genocide frequently suffer enduring psychological effects, including posttraumatic stress disorder (PTSD), complex PTSD, moral injury, depression, and anxiety disorders, which may indirectly influence their offspring through various biological, psychological, and socio-cultural mechanisms (10, 14–16).

Empirical evidence increasingly suggests that descendants of genocide survivors also exhibit heightened vulnerability to psychological disorders, particularly in high-risk environments and during new, ongoing collective traumas. Previous experiences of genocidal trauma can be reactivated by ongoing or continuous exposure to stress, shedding light on the processes that drive transgenerational trauma transmission (17, 18). The traumatic legacy may increase vulnerability in survivors and their descendants, making them more susceptible to subsequent traumatic events and potentially converting initial trauma responses into chronic patterns (19). Epigenetic mechanisms, in particular, may help explain why previously latent trauma effects emerge under renewed stress (14). Studies have demonstrated that descendants of genocide survivors – who were not directly exposed to the original trauma – may exhibit significant symptoms of PTSD, anxiety, and depression in a high-risk environment (20–22). These mental health issues imply that the traumatic consequences of genocide extend far beyond those directly exposed. This pattern is also consistent with the DSM-5 diagnostic criteria for PTSD, which recognise that learning about traumatic events occurring to a close family member or friend, particularly when those events involve violent or accidental death, can constitute trauma exposure (23). Thus, the intergenerational transfer of trauma not only highlights the importance of considering both historical and current contexts in trauma research but also aligns with current clinical definitions.

### Rationale for focusing on transgenerational transfer

Although extensive research has documented the profound psychological impacts of genocidal trauma on survivors, increasing scholarly attention has turned toward understanding how these traumatic effects may transcend the direct victims, affecting subsequent generations who were not directly exposed to the original traumatic events (16, 19). This phenomenon, commonly referred to as transgenerational (or intergenerational) trauma, suggests that descendants of trauma survivors may exhibit heightened vulnerability to psychological disorders despite the absence of direct trauma exposure. The second generation, particularly among descendants of Holocaust survivors, has received the most empirical attention, with findings indicating elevated levels of psychological distress, anxiety disorders, and PTSD symptoms compared to individuals whose parents did not experience genocidal trauma (8, 24). Moreover, recent studies increasingly highlight the importance of considering age and gender, particularly the heightened vulnerability among women, as key variables in understanding the transgenerational transfer of trauma (22). However, the role of age as a risk or protective factor in intergenerational trauma remains inconsistently presented and understudied, warranting further empirical investigation (25, 26).

A range of theoretical frameworks has been proposed to explain these patterns, underscoring the multifaceted nature of trauma transmission across generations. Psychodynamic theories highlight how unresolved trauma can disrupt family dynamics, foster a “conspiracy of silence,” and transmit unconscious fears and anxieties to offspring (11, 27). Developmental and attachment-based models further suggest that trauma may impair caregiving behaviours, erode secure attachment bonds, and increase emotional dysregulation in children, thereby contributing to mental health vulnerabilities (28–30).

In parallel, advances in molecular biology and epigenetics offer compelling evidence for the biological embedding of trauma. Research demonstrates that exposure to severe trauma, including genocide and famine, can induce lasting changes in gene expression through epigenetic mechanisms such as DNA methylation. These changes may be transmitted to offspring, influencing stress regulation, immune function, and metabolic and neurodevelopmental pathways (31–33). Studies involving descendants of Holocaust survivors and those exposed to the Dutch Hunger Winter have reported altered methylation in genes such as *FKBP5*, *NR3C1*, *IGF2*, *LEP*, and *COMT*, which are implicated in glucocorticoid sensitivity, metabolic regulation, and emotional processing (34–36).

Recent research on the long-term effects of the Holodomor has similarly indicated that exposure to famine during early gestation is associated with a more than twofold increased risk of developing type 2 diabetes later in life, underscoring early pregnancy as a critical window for shaping long-term metabolic health (37). These findings support a shared biological basis for inherited vulnerability across diverse forms of mass trauma. This growing body of evidence is further reinforced by neurocognitive and neurobiological studies showing trauma-related alterations in brain structure, functional connectivity, and stress reactivity across generations (38–41).

Furthermore, sociocultural mechanisms such as collective memory, cultural narratives, and societal stigmatisation also play a critical role in transmitting trauma across generations (11, 27, 42). Communities affected by genocide often sustain collective trauma narratives that shape cultural identity, influencing descendants’ psychosocial adjustment and mental health trajectories (10, 16, 19, 42, 43).

Despite various theoretical and empirical insights, quantitative estimates of probable PTSD prevalence in the context of transgenerational trauma remain scarce. This meta-analysis addresses this gap by systematically estimating probable PTSD prevalence among descendants of genocidal trauma. By integrating findings across psychological, biological, and sociocultural domains, the study provides a multi-level understanding of trauma transmission and may highlight potential avenues for clinical intervention in the aftermath of genocide.

### Limitations in existing literature and the need for review

While significant theoretical and empirical advancements have been made in understanding transgenerational trauma, several critical limitations persist within the current literature. Firstly, studies examining transgenerational effects of genocidal trauma have employed diverse methodologies, ranging from qualitative case studies to quantitative cross-sectional and longitudinal designs, leading to considerable variability in findings and conclusions (24, 44). This methodological heterogeneity complicates attempts to generalise outcomes across populations and genocidal contexts.

Secondly, there is considerable variability in both the conceptualisation and measurement of psychological outcomes across studies. While some investigations focus on clinical symptoms such as depression, anxiety, or PTSD, others define outcomes more broadly, encompassing coping strategies, resilience, or culturally grounded healing practices (27, 44). These conceptual differences complicate synthesis and comparison. In addition, many studies utilise psychological instruments that vary in psychometric rigor and cultural appropriateness, with some lacking validation in the populations being studied, thereby limiting the reliability of findings (45, 46).

Thirdly, previous research on different genocidal traumas has often been limited, focusing predominantly on descendants of Holocaust survivors, or on the transgenerational transfer of collective traumas, typically at the level of systematic reviews only rarely through meta-analysis (26, 47–50). Far fewer studies have systematically explored the mental health impacts among descendants of other genocidal contexts – such as the Rwandan, Cambodian, or Armenian genocides – or among those affected by genocides in Bosnia, Guatemala, Namibia, or Darfur, among others (51–53), and many have not fully accounted for key demographic variables such as age and gender. The absence of systematic control for these factors leaves open the possibility that observed psychological effects in descendants may not be solely attributable to transgenerational mechanisms (45, 54).

Finally, although narrative and qualitative reviews have contributed significantly to conceptualising transgenerational trauma, systematic quantitative syntheses (meta-analyses) remain scarce. To date, there is a notable lack of comprehensive meta-analytic studies quantifying the overall magnitude and consistency of mental health outcomes linked specifically to genocidal trauma across multiple generations and cultural contexts (44). This gap underscores the critical need for an integrative meta-analytic approach to provide statistically robust estimates of these effects.

Addressing these limitations through a systematic review and meta-analysis is therefore essential. Such an approach offers the capacity to synthesise disparate findings, quantify pooled prevalence of disorders related to genocidal trauma, identify factors moderating outcomes, and critically appraise methodological quality. Ultimately, this will enhance theoretical clarity, improve methodological standards, and inform evidence-based practices and policies tailored to the unique needs of populations affected by genocidal trauma across generations.

### Study objectives and research questions

In light of the above limitations and theoretical considerations, the present systematic review and meta-analysis aim to examine the transgenerational transfer of genocidal trauma among offspring, considering cross-cultural variations.

To achieve the objective, this systematic review and meta-analysis will be guided by the two research questions:

RQ1: What is the overall prevalence of PTSD among offspring of genocide survivors across different cultural settings?

RQ2: Are age and female gender associated with increased risk for PTSD in the descendants of genocide survivors?

## Method

The protocol for the systematic review and meta-analysis was pre-registered on PROSPERO (CRD420251046525) on the May 8, 2025. We have conducted a systematic search of Medline (via PubMed), PsycINFO (via EBSCOhost), and PTSDpubs (via ProQuest) for empirical studies published in English between January 2000 and April 2025. Eligible sources included peer-reviewed journal articles, doctoral and master’s theses, and preprints.

Search terms were (“Trauma and Stressor Related Disorders”[Mesh] OR “Stress Disorders, Post-Traumatic”[Mesh] OR “Psychological Trauma”[Mesh] OR “Post-Traumatic Stress Disorder”[tw] OR PTSD[tw]) AND (“Genocide”[Mesh] OR “Holocaust”[Mesh] OR genocide[tw] OR holocaust[tw] OR “Rwandan genocide”[tw] OR “mass atrocity”[tw]) AND (descendants[tw] OR offspring*[tw] OR “second generation”[tw] OR “third generation”[tw] OR “fourth generation”[tw] OR children[Mesh] OR children[tw] OR “parent-child relations”[Mesh] OR “parent-child relations”[tw]) AND (“Mental Health”[Mesh] OR “Mental Disorders”[Mesh] OR “Psychological Effects”[tw] OR “Psychosocial impact”[tw] OR “Psychological consequences”[tw] OR “mental health outcomes”[tw]) for Medline (via PubMed).

Search terms for PsycINFO (via EBSCOhost) were “Transgenerational trauma” OR “Intergenerational trauma” OR “Genocidal trauma” OR “Historical trauma” OR “Collective trauma” Or “Mass Atrocity” AND “Post-Traumatic Stress Disorder” OR PTSD AND “Genocide” OR “Holocaust” OR “Rwandan genocide” AND “Descendants” OR “Second/third/fourth generation” OR “Children” OR “Offspring” AND “Psychological effects” OR “Mental health outcomes” OR “Psychosocial impact” OR “Psychological Consequences”.

### Searching process

Studies have been included if they: (1) used cross-sectional, longitudinal, cohort designs; (2) assessed prevalence of PTSD with possible (but not necessary) reports of other mental health outcomes (e.g. depression, anxiety, trauma-related mental health symptoms) in descendants of genocide survivors using validated psychometric instruments; (3) report on second, third, or subsequent generations exposed indirectly to genocidal trauma; (4) papers written in English and published in peer review journals or doctoral and master’s dissertations/theses on PTSDpubs (ProQuest), or preprints. Studies that do not meet these criteria have been excluded if no further information can be obtained from the authors.

In longitudinal studies, point prevalence of PTSD has been assessed at baseline, allowing us to examine the prevalence at a specific time. To ensure the reliability of the search strategy, a validation step was conducted using a reference set of two previously known articles, all of which were selected from PTSDpubs. Searches were carried out across three major databases: Medline (via PubMed), PsycINFO (via EBSCOhost), and PTSDpubs (via ProQuest). One researcher (LZ) cross-checked the search results against those retrieved from Research4Life, an academic access platform supporting research and education in low-resource settings. In July 2025, the search was repeated to capture any newly published studies that might meet the inclusion criteria; and no eligible papers were identified at that time.

### Screening process

**Figure 1:**
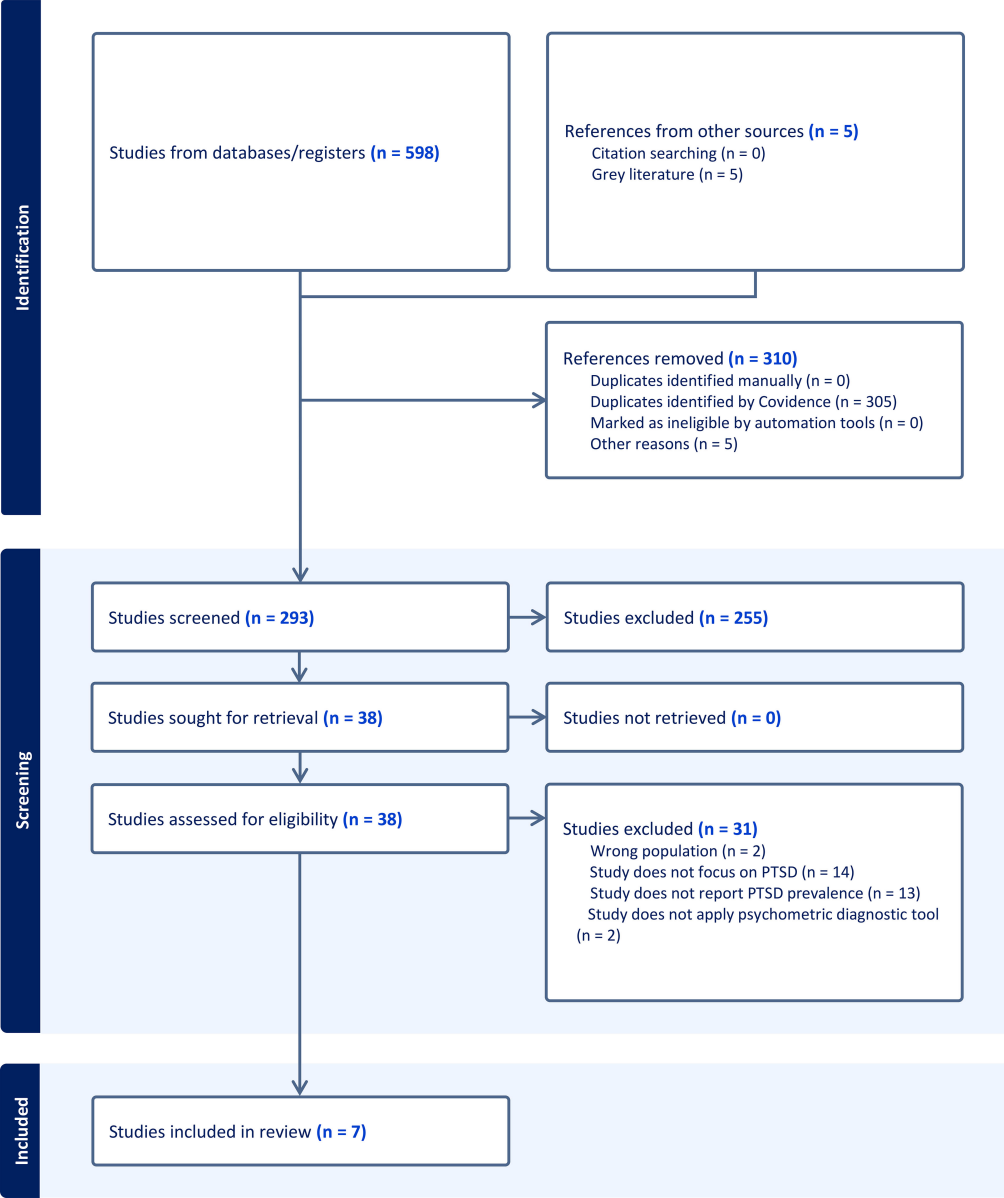
PRISMA flow illustrating study selection and exclusion process.

Study selection and screening were managed using *Covidence*, a web-based platform designed for systematic review workflows, including blinded screening and data extraction. Two authors (IH and OS) independently conducted the initial screening of titles, abstracts, and keywords based on predefined inclusion and exclusion criteria. Duplicates were removed automatically and manually verified. The first author (LZ) participated in resolving any discrepancies flagged in the “Resolve Conflicts” stage. Full texts of potentially eligible studies were then independently assessed by IH, IK, OS, and LZ. Ultimately, only seven studies met all eligibility criteria. The PRISMA flow diagram illustrates the study selection process (see **Figure 1**).

### Data extraction

A data extraction form based on a Covidence template was developed, including the variables to be collected and clear definitions for them. The first author (LZ) pilot-tested the form using ten randomly selected studies to increase the reliability of the data extraction process.

Afterwards, four authors, IH, IK, OS, and LZ, independently extracted data from the included studies. They summarised the number of participants, their age, the percentage of males and females in the sample, the overall prevalence of PTSD and trauma-related disorders, the diagnostic assessment tools used, the country in which the genocide occurred, the World Bank country classification based on participants’ current place of residence, and the type of generation (first, second and subsequent generations).

### Quality of studies

The quality of each study was assessed using a risk of bias tool adapted by Woolgar et al. (55) from the Joanna Briggs Institute (Prevalence Critical Appraisal Tool, PCAT) (56). The PCAT included six questions and assessed the description of the participants and settings; participation rate of the eligible participants; reasons for non-response; quality and representativeness of the sample; appropriateness of recruitment; exclusion and inclusion criteria. Authors IH and OS allocated each study a risk-of-bias score (9-12=low risk of bias, 5-8=medium risk, 0-4=high risk). Further, each study was rated high (0), medium (1) or low (2) risk of bias on each criterion. Item-level interrater agreement between the two raters was high (Kappa = .704, p <.001). Discrepancies were discussed and resolved. Individual study ratings for each risk-of-bias criterion are detailed in **Supplementary Table S1** in Supplement 1. All studies included in the meta-analysis were assessed as having a medium level of risk of bias using PCAT tool, therefore, risk of bias was not used as a potential moderator in subsequent analyses.

### Statistical analysis

We conducted meta-analyses using the metafor package in R (57). Mixed-effects logistic regression models were employed to appropriately analyse count and prevalence data (58). Our results include estimates of heterogeneity (τ²), inconsistency (I²), and prediction intervals, which describe the expected variation in prevalence across studies and are recommended for meta-analyses of proportions (59). Notably, the statistics I² and H², which can typically be calculated from published meta-analyses, offer particularly valuable summaries of the impact of heterogeneity. In line with current best practice, one or both of these measures should be reported in preference to relying solely on the test for heterogeneity, as they provide more informative insights into the variability and reliability of effect size estimates across studies (60).

First, a model without moderators was fitted to estimate the overall prevalence of PTSD. To investigate potential moderators (gender and age), we introduced study-level variables individually into the models. The study applied the rma() function from the metafor package, which automatically transformed proportions and computed heterogeneity estimates. We reported the omnibus test of moderation significance (Qm test), which evaluates the null hypothesis that probable prevalence rates do not differ across moderator groups.

To assess the potential influence of gender on PTSD prevalence, we included the percentage of female participants in each study as a continuous, study-level moderator in a meta-regression model. This approach allowed us to examine whether the proportion of females in a given study sample was associated with reported PTSD prevalence rates. Due to the limited reporting in the selected papers, sex and gender were treated as equivalent in this analysis.

## Results

The primary genocidal traumas represented in the seven finally selected studies with a total of 1,569 participants were originated from major historical genocides, including those in Cambodia (61), Armenia (62), Rwanda (53, 63–65), and Israel (50) see **Table 1**. These countries differ significantly in terms of their World Bank income classifications, with Rwanda and Cambodia classified as low- or lower-middle-income countries, Armenia as upper-middle-income country, and Israel as a high-income country. This variation in economic context may contribute to differences in trauma exposure, access to mental health resources, and PTSD prevalence across studies. Most studies employed purposeful sampling, with Burchert et al. (61) and Shrira et al. (50) being the only two to use random sampling.

**Table 1:** Studies included in the meta-analysis.

Author	Year	Age-range	Age-mean (SD)	Country	Sample size	Proportion of females (%)	Probable PTSD prevalence (%)	Diagnostic assessment tool
Burchert et al. (61)	2017	18 ≥	23.4 (4.0)	Cambodia	378	55	8.5	PCL-C
Haladjian (62	2020	18-75	46.61 *	Armenia	129	67.4	21.3	MSTS
Ingabire et al. (63)	2023	17-24	20.37 (1.78)	Rwanda	181	59	9.00	PCL-5
Mutuyimana et al. (64)	2019	14-22	17.38 (2.02)	Rwanda	432	60.88	16.5	PCL-5, UCLA-PTSD-RI
Rudahindwa et al. (65)	2020	–	17.5*	Rwanda	25	52	36	PCL-17
Shrira et al (53).	2019	–	21.21 (1.78)	Rwanda	60	46.67	37.3	PDS-ICD-11
Shrira et al. (50)	2025	–	44.07 (13.80)	Israel	364	49.2	10.4	ITQ

Note: *Missing standard deviation. **Missing mean and standard deviation. Acronyms of measures are as follows: PCL-C, The PTSD Checklist – Civilian Version; MSTS, Modified Secondary Trauma Scale; PCL-5, PTSD Checklist for DSM-5; PDS, Posttraumatic Diagnostic Scale; UCLA-PTSD-RI, The University of California Los Angeles Post-traumatic Stress Disorder Reaction Index; PDS-ICD-11, Personality Disorder Severity ICD-11; ITQ, International Traumatic Questionnaire; PCL-17, PTSD Checklist with 17 items.

All studies were evaluated as having a medium risk of bias. Although some studies included data from three (survivors, their children and grandchildren) (50), and four (62) generations, all of them provided data on second-generation participants. Therefore, we focused our analysis on trauma-related mental health outcomes in this population.While most studies emphasised trauma-related disorders, Rudahindwa et al. (65) and Shrira et al. (50) examined perceived parental or grandparental probable PTSD and its association with second-generation outcomes.

A random-effects meta-analysis was conducted on seven studies to estimate the pooled prevalence of PTSD. The analysis employed the restricted maximum likelihood (REML) estimator to account for between-study heterogeneity.

**Figure 2:**
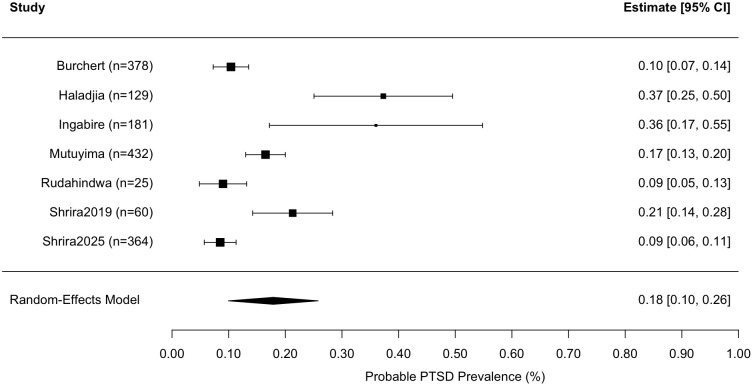
Forest plot of probable PTSD prevalence rates for each study and meta-analysis results of probable PTSD rates and 95% prediction intervals. Note. The black diamond shows the 95% confidence interval for the meta-analytic average prevalence rate, and the error bars show the 95% prediction intervals, which are much broader due to heterogeneity between studies.

The estimated total heterogeneity (τ²) was .010 (SE = .007), indicating substantial variability in prevalence estimates across studies. This was supported by a high I² value of 95.03%, suggesting that approximately 95% of the total variability in effect sizes was due to true heterogeneity rather than sampling error. The test for heterogeneity was statistically significant, Q(6) = 44.61, p <.001, confirming that the included studies were not homogeneous. The pooled effect size estimate on the proportion scale was .179 (SE = .041), which was statistically significant (z = 4.42, p <.0001). After back-transformation, this corresponds to an estimated pooled PTSD prevalence of approximately 17.9% (95% CI: 9.9% - 25.8%) (see **Figure 2**).

These findings point to a notably high prevalence of PTSD across the included studies, accompanied by considerable variation between study estimates. The observed heterogeneity may be attributed to the diverse sociopolitical contexts in which the studies were conducted. Several studies were based in regions experiencing ongoing or recent armed conflict, such as Israel, Cambodia, Rwanda, and Armenia, where populations are at increased risk of war-related trauma and chronic stress. Moreover, socioeconomic factors, including low income and limited access to mental health services in these settings, likely contributed to both elevated PTSD prevalence and between-study variability. Additionally, heterogeneity in PTSD prevalence may be increased by the use of different diagnostic assessment tools across studies, including four studies (61, 63–65) that employed the PTSD Checklist (PCL) based on DSM criteria. Others used alternative measures; in particular, Shrira et al. (50, 53) applied the ICD-11 PTSD and the International Trauma Questionnaire (ITQ), which assesses PTSD and Complex PTSD, and Haladjian (62) employed the Modified Secondary Trauma Scale (MSTS). The MSTS consists of 18 items derived from the six PTSD criteria outlined in the DSM-IV (23), thus reflecting a secondary conceptualisation of trauma but still grounded in standard diagnostic framework for PTSD.

Across seven studies, participant characteristics varied widely, with a mean age of 31.73 years (SD = 16.14) with an age range of 17–55 and an average female representation of 51.22% (SD = 14.23%). The unweighted average PTSD prevalence across these studies was 19.9% (SD = 12.4%), with individual study estimates ranging from 8.5% to 37.3%. For instance, the study by Shrira et al. (53) reported the highest PTSD prevalence (37.3%) among a relatively young sample (mean age 21.2 years; 46.7% female), with 26.7% of their parents suffering from PTSD and 33.3% having Complex PTSD. In comparison, Rudahindwa et al. (65) reported a 36% PTSD prevalence among offspring whose mothers were pregnant during the genocide in Rwanda. This variation in sample demographics and measurement methods highlights the complexity of synthesising PTSD prevalence across diverse populations.

**Figure 3:**
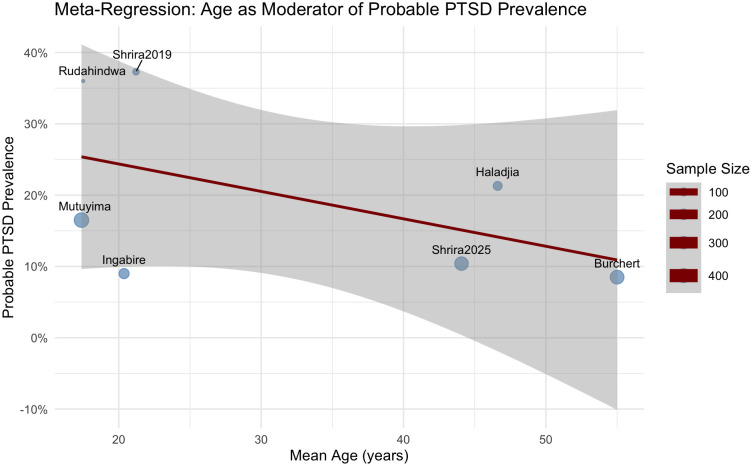
Bubble plot of study age and the probable PTSD prevalence rates. Note. The size of each point is proportional to the precision (inverse of variance) of the effect size estimate, meaning that studies with larger sample sizes and more precise estimates are represented with larger points.

To explore potential moderators of probable PTSD prevalence across studies, we conducted separate mixed-effects meta-regression models using age and female proportion as continuous, study-level predictors see **Table 2**. A mixed-effects meta-regression model including mean age as a moderator was conducted across seven studies. The model did not explain a significant proportion of heterogeneity in PTSD prevalence, *QM* (1) = 2.91, *p* = .256. The regression coefficient for age was not statistically significant, (b = -.023, SE = .018, z = -1.263; p = .207, 95% CI [-.059, .013]. This indicates that variations in average participant age across studies were not significantly associated with probable PTSD prevalence rates. Residual heterogeneity remained substantial, *τ²* = .471 (SE = .336), *I²* = 90.91%, suggesting that a large proportion of variability across studies was not accounted for by age (see **Figure 3**).

**Table 2:** Meta-regression results testing which factors moderate the probable PTSD prevalence rates across studies.

Variable	b	95% confidence interval	
		Lower bound	Upper bound
Mean age (years)	QE​ (df=5) =43.039, p<.0001 QM ​(df=1) =.595, p=.207		
Intercept	-1.577	-2.116	-1.037
Age-centered	-.023	-1.263	.207
Female	QE(df = 5) = 44.925, p <.0001 QM(df = 1) = .434, p = .510		
Intercept	b = -1.583	b = -2.175	b = -.992
Female (centered)	b = 1.483	b = -2.930	b = 5.895

Note: QE, Test for Residual Heterogeneity; QM, Test of Moderators.

**Figure 4:**
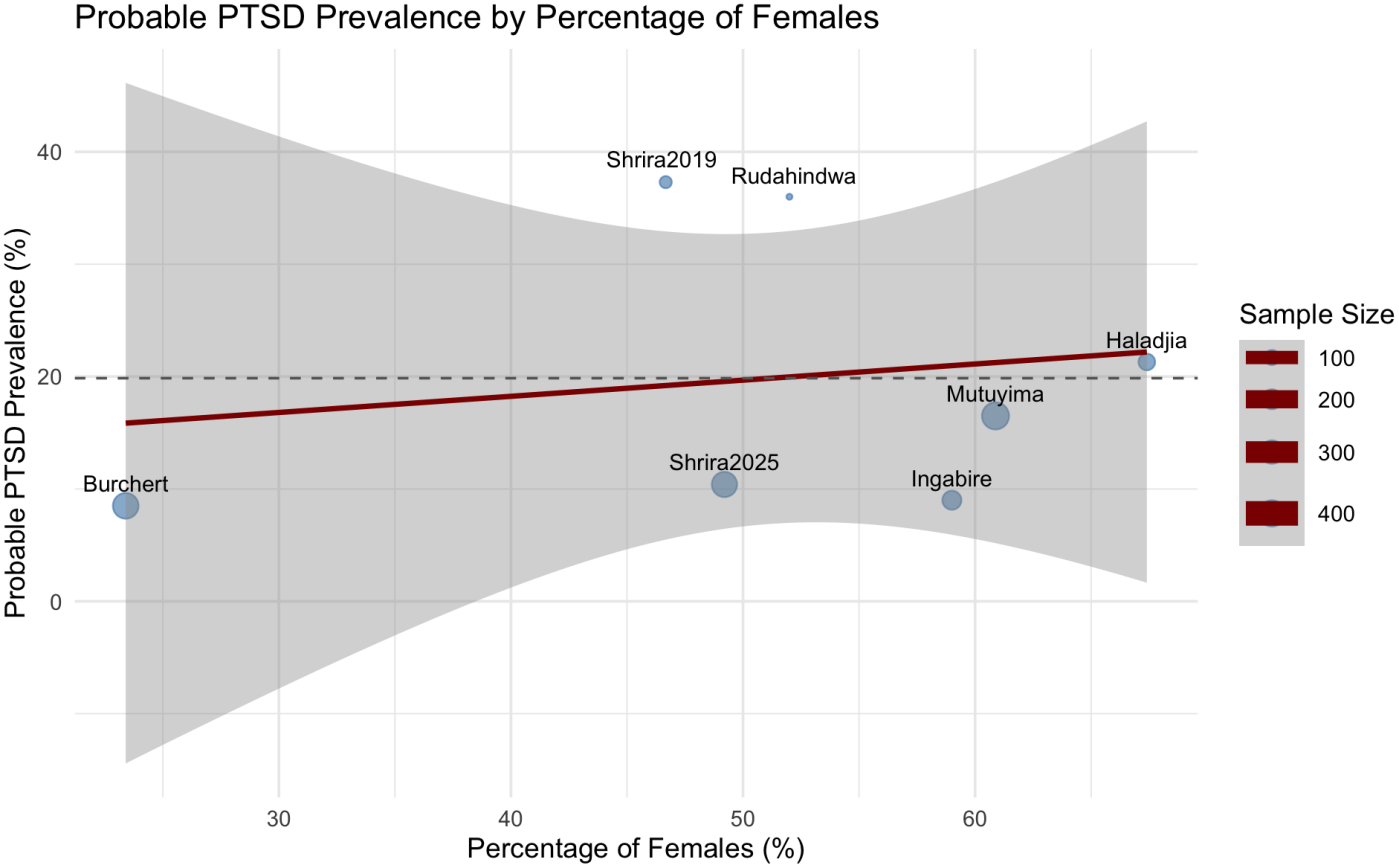
Bubble plot of study female gender and the probable PTSD prevalence rates. Note. The size of each point is proportional to the precision (inverse of variance) of the effect size estimate, meaning that studies with larger sample sizes and more precise estimates are represented with larger points.

We next examined whether the proportion of female participants in each sample predicted probable PTSD prevalence. This model, based on seven studies, similarly did not yield a significant moderation effect, QM(1) = .434, p = .510. The regression coefficient for female proportion was non-significant, b = 1.483, SE = 2.251, z = .659, p = .510, 95% CI [-2.930, 5.895], suggesting that the percentage of female participants in the study samples was not significantly associated with probable PTSD prevalence. Residual heterogeneity remained high in this model, with τ² = .578 and I² = 92.77% (see **Figure 4**).

These findings suggest that differences in age or gender composition could not explain variability in probable PTSD prevalence across studies.

## Discussion and conclusions

This systematic review and meta-analysis of seven studies examining trauma-related mental health outcomes in second-generation descendants of genocide survivors reveals a notably high pooled PTSD prevalence of approximately 17.9%, with individual study estimates ranging from 8.5% to 37.3%. Despite high heterogeneity across studies, the overall effect was statistically significant. Mixed-effects meta-regressions showed that neither mean age nor proportion of female participants significantly predicted probable PTSD prevalence, with both models leaving a large proportion of variance unexplained (I² > 90%). These findings underscore the enduring psychological toll of genocidal trauma across generations and suggest that factors beyond demographic composition, including sociopolitical context and methodological variation, may cause differences in PTSD prevalence.

The included studies spanned a diverse range of genocidal contexts, including those in Cambodia, Armenia, Rwanda and Israel, reflecting historical traumas of varying scales and temporal distances. All studies focused on second-generation participants, although some also included third and fourth generations (50, 62). The emphasis on second-generation outcomes allowed for a more consistent comparison across studies.

According to our first research question, the findings from this meta-analysis indicate a pooled PTSD prevalence of 17.9%, which is substantially higher than the global lifetime prevalence of PTSD. Recent studies indicate that varying rates of PTSD typically range between 1% and 12%, depending on the population and context (66). In the United States, the estimated prevalence of PTSD is 4.7% over the past year and 6.1% across the lifetime (67). Although self-report measures may yield different estimates compared to structured clinical interviews, the PCL, as noted by Merians (67), remains a reliable and valid instrument for assessing PTSD symptoms in non-psychiatric or community settings. Its use in the four included studies therefore underscores the importance of capturing trauma-related distress in broader populations.

Importantly, the observed prevalence in second-generation offspring of genocide survivors is not substantially lower than rates reported among civilian populations during active military conflict or in its aftermath. In our previous study examining PTSD among civilian youth exposed to war, an estimated PTSD prevalence is 29.4% (68). These results underscore the enduring psychological burden transmitted across generations in the aftermath of genocidal trauma, which appears to persist decades after the original traumatic events.

The heightened prevalence may be further amplified by the politically unstable contexts in which several of the selected studies were conducted. These contexts can reactivate unresolved trauma and contribute to the accumulation of new traumatic stressors. Notably, the inclusion of low-income and low-middle-income countries (LMIC) such as Rwanda and Cambodia, where resources for mental health care remain scarce, may intensify this effect by limiting opportunities for recovery and increasing vulnerability. Despite the heightened need in LMIC, treatment-seeking rates there remain disproportionately low, particularly in low- to lower-middle-income countries. Treatment-seeking has been estimated at 22.8% in these countries, compared to 28.7% in upper-middle-income countries and 53.5% in high-income countries (69). Together, these findings show that genocidal trauma is not merely a residual psychological effect but a significant public mental health concern, particularly in sociopolitically disadvantaged settings.

Findings of the current research indicate that precise estimation of PTSD prevalence remains difficult due to the high heterogeneity across studies, which may be explained not only by differences in political contexts but also by variability in diagnostic assessment tools, sample sizes, and methods of participant recruitment. These methodological and contextual differences can influence prevalence estimates and impede comparability across settings. The selected studies employed diagnostic tools grounded in both the DSM and ICD frameworks, reflecting variation in how PTSD is measured across research contexts (68, 70). This highlights the critical need for employing validated and culturally sensitive assessment instruments. Future research aimed to develop PTSD assessment tools that reflect both global diagnostic standards and cultural-linguistic concepts of trauma expression is needed.

Notably, there is a relative scarcity of quantitative studies focused on the prevalence of PTSD among offspring of genocide survivors, with only seven studies meeting the criteria for inclusion in this meta-analysis. This reflects a broader trend in the literature, where most research has focused on qualitative approaches aimed at uncovering intergenerational mechanisms rather than quantifying trauma-related mental health outcomes. Consequently, meta-analytic approaches are essential to synthesise available data and provide evidence-based understanding of genocidal trauma prevalence across diverse settings.

In relation to the second research question, the non-significant results from the meta-regressions, with age and gender as potential moderators, suggest that demographic factors alone do not sufficiently account for the variation in probable PTSD prevalence across studies. These findings diverge from conclusions in qualitative research that emphasised children’s vulnerability and female-specific experiences of genocidal trauma (71, 72). However, our results should not be interpreted to mean that gender differences in trauma impact do not exist. Instead, the lack of significant findings may stem from the limited number of included studies reporting the necessary quantitative data.

Moreover, the influence of demographic characteristics may be mediated or overshadowed by more complex psychosocial and contextual moderators. Factors such as patterns of familial communication about trauma, community-based support systems, cultural stigma around mental health, and even biological mechanisms such as epigenetic modifications may play a more substantial role in shaping vulnerability or resilience to transgenerational trauma than age or gender alone (20, 22, 73, 74). These findings point to the need for more multi-layered research of risk and protective factors that go beyond demographic descriptors.

An important and still underexplored area of research is whether the mental health consequences of genocidal trauma differ from those following other forms of trauma, including unintentional traumas as natural disasters, interpersonal traumas as ongoing violence, or intentional collective traumas such as armed conflicts and wars. Understanding these differences is vital for tailoring interventions and support systems to genocidal trauma legacies. This gap highlights the need for future research on trauma-related mental health conditions among different generations of descendants, using culturally sensitive diagnostic assessment tools to understand better the interplay between PTSD and comorbidities of genocidal trauma.

## Limitations

Due to the small number of studies, the analyses were exploratory in nature and primarily aimed at generating hypotheses for future research with a larger dataset. The instruments used to assess probable PTSD rates varied across studies, which further limited the consistency required for robust meta-regression with multiple moderators. Given these constraints, we selected gender and mean age as the most consistently reported and theoretically relevant study-level moderators. These variables are well-documented demographic factors known to influence PTSD prevalence and presentation, and they offered sufficient variability across studies to warrant exploratory analysis. While other potential moderators, including diagnostic assessment tool, socioeconomic context, type of generation and risk of bias, may also contribute meaningfully to differences in PTSD prevalence, the current dataset lacked the breadth and variability necessary to examine these factors reliably.

Importantly, both participant age and the time elapsed since the genocide may play key roles in shaping the transfer of trauma-related symptoms. In the current meta-analysis, the time elapsed since the genocide was inconsistently reported across studies, limiting our ability to include this variable as a moderator. Participant age may not always serve as a reliable proxy for the temporal distance from the genocidal trauma, highlighting the need to consider both age and the aftermath period of genocidal trauma as parallel moderators in future research.

To assess the potential influence of gender on PTSD prevalence, we used the percentage of female participants in each study as a study-level continuous moderator in a meta-regression model. This approach allowed us to test whether studies with a higher proportion of female participants were associated with increased PTSD prevalence. As a result, our analysis could only capture study-level associations rather than individual-level risk differences. This limits our ability to draw causal or directional conclusions about gender as a risk factor for PTSD. Furthermore, many of the included studies did not clearly differentiate between biological sex and gender identity, nor did they report how gender was measured. This introduces additional ambiguity and reflects a broader limitation in the literature on trauma exposure and gendered outcomes. Future meta-analyses would benefit from access to disaggregated participant-level data and more consistent reporting of gender and sex.

Only two studies in the meta-analysis employed random sampling, while all others used purposive recruitment methods. This may introduce selection bias and limit the generalisability of the pooled PTSD prevalence estimates in offspring of genocide survivors. Although we did not control for recruitment method in the meta-analytic models, we used the PCAT tool to systematically assess study quality and risk of bias, including the item “Were participants recruited in an appropriate way?” (see **Supplementary Table S1**). This allowed us to document potential sources of bias across studies.

Additionally, a significant limitation of the current meta-analysis lies in the timing and nature of data collection. Most studies assess the mental health of direct descendants years or even decades after the genocide occurred, meaning that other post-genocide stressors, such as poverty, discrimination, and political instability, may accumulate and influence outcomes. Since meta-analyses synthesise study-level data rather than individual-level trajectories, they are limited in their ability to control for such cumulative effects. This methodological constraint further underscores the need for longitudinal, culturally grounded, and contextually sensitive studies that can better explore the specific impact of genocidal trauma separately from subsequent life adversities.

## Data Availability

The datasets presented in this study can be found in online repositories. The names of the repository/repositories and accession number(s) can be found below: https://github.com/LZasiekina/Transgenerational-Transfer-of-Genocidal-Trauma-A-Systematic-Review-and-Meta-Analysis.git.
