# Supplementary material for: Transgenerational transfer of genocidal trauma: a systematic review and meta-analysis
Source: Front Psychiatry. 2026 Jan 30;16:1699835. doi: 10.3389/fpsyt.2025.1699835 (PMC12901391; doi:10.3389/fpsyt.2025.1699835)
Supplement: Supplementary file 1 [file Supplementaryfile1.docx]

Supplement 1

Table S1

| Paper | Were participants and settings well described? | Was the participation rate of those eligible at least 50%? | Were reasons for non-responders described? | Was the sample representative – were there differences between those participants taking part and those not?? | Were participants recruited in an appropriate way? | Were inclusion and exclusion criteria explicit and appropriate? | Score 0/12 | Score 0/2 |
| --- | --- | --- | --- | --- | --- | --- | --- | --- |
| Burchert et al, 2017 | 2 | 0 | 0 | 0 | 2 | 2 | 6 | 1 |
| Haladjian, 2021 | 2 | 0 | 1 | 0 | 1 | 2 | 6 | 1 |
| Ingabire et al, 2023 | 2 | 0 | 0 | 0 | 2 | 2 | 6 | 1 |
| Mutuyimana et al, 2019 | 2 | 1 | 0 | 0 | 1 | 2 | 6 | 1 |
| Rudahindwa et al, 2020 | 1 | 0 | 0 | 0 | 2 | 2 | 5 | 1 |
| Shrira et al, 2019 | 2 | 1 | 1 | 0 | 1 | 2 | 7 | 1 |
| Shrira et al, 2025 | 2 | 1 | 1 | 0 | 1 | 2 | 7 | 1 |

Note. 9-12=low risk of bias, 5-8=medium risk, 0-4=high risk. Further, each study was rated high (0), medium (1) or low (2) risk of bias on each criterion.
